# Effectiveness of work-based educational interventions for antimicrobial stewardship: a systematic review

**DOI:** 10.1093/jacamr/dlae192

**Published:** 2024-12-10

**Authors:** Darren Langdridge, Jennika Virhia, Rachel McMullan, Duncan Banks, Olivier Biard, Koula Charitonos, Jimmy Patrick Alunyo, Enid Kawala Kagoya, Peter Olupot-Olupot

**Affiliations:** School of Psychology and Counselling, The Open University, Milton Keynes MK7 6AA, UK; School of Biodiversity, One Health and Veterinary Medicine, University of Glasgow, Glasgow, UK; School of Life, Health and Chemical Sciences, The Open University, Milton Keynes, UK; School of Life, Health and Chemical Sciences, The Open University, Milton Keynes, UK; Global Development Team, The Open University, Milton Keynes, UK; Institute of Educational Technology, The Open University, Milton Keynes, UK; Department of Research, Mbale Clinical Research Institute, Mbale, Uganda; Department of Community and Public Health, Busitema University, Busitema, Uganda; Department of Community Health, Busitema University, Busitema, Uganda; Clinical Epidemiology Unit, School of Medicine, Makerere University, Kampala, Uganda; Department of Research, Mbale Clinical Research Institute, Mbale, Uganda; Department of Community and Public Health, Busitema University, Busitema, Uganda

## Abstract

**Background:**

The pressing need for better antimicrobial stewardship (AMS) is invariably reliant on educational interventions in some form.

**Objectives:**

To evaluate the effectiveness of post-qualification educational interventions for AMS behaviour change among health professionals.

**Methods:**

Seven databases were searched for articles published between 2013 and 2024 for post-qualification educational interventions aimed at health professionals to improve AMS. Randomised controlled trials (RCTs) and quasi-experimental designs such as non-randomised trials, controlled and non-controlled before and after studies, and qualitative studies were considered eligible. The quality of studies was assessed using Cochrane Effective Practice and Organization of Care (EPOC) criteria for RCTs and interrupted time series designs, and the Mixed Methods Appraisal Tool (MMAT) for all other studies. Data were extracted, analysed for effectiveness, and synthesised narratively. Registration: PROSPERO international prospective register of systematic reviews (PROSPERO 2023 CRD42023447115).

**Results:**

Forty-six studies were included in the review, with six meeting the EPOC criteria. The remaining forty were assessed using the MMAT. The overall risk of bias for the six studies meeting the EPOC criteria was low, but risk of bias was high for studies assessed using the MMAT. Overall, there was some evidence that formal education alone was effective in this context, but only limited evidence about what type of educational intervention, for which profession, is most effective.

**Conclusions:**

Our review provided an in-depth examination of post-qualification AMS interventions. We found studies were heterogeneous and quality of evidence relatively poor. High quality studies focused on establishing key components of effective educational interventions are required.

## Introduction

The rise of antimicrobial resistance (AMR) continues to present a global public health threat. It is estimated that by 2050, AMR will be responsible for around 10 million global deaths each year, with the greatest burden of mortality being in low-resource settings (LRSs).^1^ For example, recent estimates suggest that, in sub-Saharan Africa, 75 of 100 000 deaths were directly associated with AMR in 2019.^2^ With such dire predictions of AMR’s future growth across the globe and with LRSs being more vulnerable to its spread and impacts, AMR will further widen global health inequalities, threatening every aspect of medicine and healthcare.

A key contributor to the spread and development of AMR is the use of antibacterial drugs when not strictly necessary. This gives bacteria the opportunity to develop resistance and survive the effects of antibacterial drugs, making them ineffective. Antimicrobial stewardship (AMS) is now widely accepted as a systematic approach for health professionals to enhance and monitor the appropriate use of antimicrobials,^3,4^ in order to curb resistance. The concept has been identified globally in the WHO’s Global Action Plan for AMR, as well as nationally in AMR National Action Plans (NAPs) as a key strategic priority for addressing resistance. Gaps in understanding and awareness related to AMR/AMS among professionals in the health and veterinary sectors and agricultural practice have also been identified. Making AMR a key component of professional education, training, continuing education and development will thus help to address this. Particularly, the education and training of health professionals in stewardship practices is vital for safeguarding the effectiveness of antibacterial drugs. However, due to changing AMR-related knowledge and skills, with professional roles evolving rapidly,^5^ it is critical to provide health professionals with regular education to develop and apply new AMR-related knowledge and skills, as well as building capacity in support of systemic change.

However, little evidence exists on the way learning related to AMS is enabled in professional work roles and settings, and specifically on the type, mode and delivery of professional education and learning that is likely to result in sustained behaviour change that can contribute to effective stewardship: that is, what educational interventions work and for whom? Approaches traditionally used in professional education and learning in the workplace, such as one-off formal Continuing Professional Development (CPD) sessions, often become synonymous with participation in courses or seminars and are often insufficient to bring about effective and lasting behaviour change. Such approaches can obscure the links between formal learning and the context of everyday practice.^6^ Furthermore, the knowledge acquired in CPD events is often supposed to be ‘transferred’ to the workplace but may overlook prevailing work-related barriers that prevent this from happening.^7^ As a result, professional education can become disconnected from professional practice when it is founded on an underlying assumption that practice can be changed or updated solely by acquisition of knowledge independent of the work context.^5^ Whilst professional education and learning within the workplace is underpinned by the notion that human learning takes place in the interface between individual and collective action,^8^ various approaches, particularly related to technology-supported learning within the workplace, tend to focus almost exclusively on individual learning, whilst eliding the fact that learning is embedded in broader social, environmental and political processes that affect behaviour.

The aim of this review is to provide best quality evidence of what work-based education interventions are likely to be effective for AMS behaviour change. In order to achieve this, we systematically examine published research on work-based learning interventions to better understand ‘what works’ in the context of AMS among post-qualification health professionals. Key to this will be a sensitivity analysis and examination of relative effectiveness. This will allow us to describe the nature of education interventions, what they involve, and what outcomes they elicit, as well as identifying gaps and areas for improvement.

## Methods

This review was prospectively registered on the PROPSERO international prospective register of systematic reviews (https://www.crd.york.ac.uk/prospero/display_record.php?ID=CRD42023447115) and is reported in accordance with the Preferred Reporting Items of Systematic Reviews and Meta-Analyses (PRISMA) statement.^9^

### Search strategy

MEDLINE, SCOPUS, Embase, CINAHL, Web of Science, CENTRAL and PsychINFO databases were searched for articles published between 2013 and 2024 using keywords associated with the following four areas: (i) population—health professionals in receipt of post-qualification learning interventions on AMS; (ii) intervention—a work-based learning intervention designed to improve AMS for human health; (iii) context—post-qualification work-based learning to improve AMS in relation to human health; (iv) outcomes—all relevant short-, medium- or long-term outcomes related to AMR and/or AMS behaviours (knowledge/awareness, learning, changed behaviour, prescribing practices). The search strategy utilised controlled terms (e.g. MeSH) where relevant, and free text within titles and abstracts. The search strategy was amended according to the functionality of each database. In addition to systematic database searching, the reference lists of included papers and previous systematic reviews were searched manually in order to identify any additional records.

### Study selection

All studies that met the review’s eligibility criteria (Table 1) were included. This included a range of study designs: randomised controlled trials (RCTs) and quasi-experimental designs such as non-randomised trials, controlled and non-controlled before and after studies, and qualitative studies. Studies pertaining to animal health, the general public, pre-qualification or that were not available in English were excluded.

**Table 1.** Review’s eligibility criteria

	Inclusion criteria	Exclusion criteria
Design	Randomised controlled trials, interrupted time series studies and quasi-experimental designs such as non-randomised trials, controlled before and after studies, non-controlled before and after studies and qualitative studies	—
Population	Health professionals in receipt of post-qualification work-based learning interventions on antimicrobial stewardship (AMS)	Members of the public
Intervention	A post-qualification work-based learning intervention designed to improve AMS for human health	—
Comparator	Time bound or geographical controls or no exposure	—
Context	Post-qualification work-based learning to improve AMS in relation to human health	—
Outcomes	All relevant short-, medium- or long-term outcomes related to AMR and/or AMS behaviours (knowledge/awareness, learning, changed behaviour)	—
Publication date	Published during or after January 2013	Published before January 2013

All educational interventions on AMS targeting post-qualification health professionals were included. We focused solely on antibacterial resistance, with studies focused on antifungal, antiviral and antimalarial stewardship and TB excluded given they involve very different contexts. Educational interventions that were concomitant with other non-educational interventions, or multi-component interventions were excluded unless the educational aspect was independently evaluated. Education proved difficult to define and there was considerable inconsistency in what was understood as education within the studies. Audit and feedback and guideline implementation were excluded, as were interventions consisting of ongoing practice innovations such as software applications designed to assist prescribing decision-making, with this study focused solely on formal education where interventions involved a prescribed learning framework or an organised learning event or package, usually involving a teacher or trainer (see Eraut^10^; and see also limitations).

All titles and abstracts of identified articles were screened against the eligibility criteria by two of three reviewers (J.V., D.L., and R.M.). J.V. screened 100% of records, while D.L. and R.M. screened 50% each. Full texts of papers that met the inclusion criteria were screened by all three reviewers. Disagreements were resolved via discussion with all reviewers.

### Data extraction and quality assessment

One reviewer (J.V.) independently extracted data from eligible studies using a standardised tool designed for the purpose of the study, resulting in inclusion of 46 studies (Table 2).^11–56^ Six studies met the EPOC design (i.e. randomized controlled trials, non-randomized controlled trials, controlled before-and-after studies and interrupted time series studies) and were assessed using EPOC risk of bias criteria.^19,26,36,47,49,56^ Forty studies did not meet the EPOC criteria (i.e. non-controlled before-and-after studies) and were assessed using the MMAT. Studies assessed with the MMAT tool included: quantitative non-randomised studies (*n* = 37); qualitative studies (*n* = 2); and mixed-methods studies (*n* = 1). Quality assessment for all studies was checked by a second reviewer (D.L.) and disagreements were resolved through consensus.

**Table 2.** Study characteristics and primary results

Study	Country	Design	Sample	Nature of intervention	Outcome measures	Significant results
Abu-Ajaleh *et al.* (2023)^11^	Jordan	Non-controlled before-and-after study	117 specialists, consultants, and resident doctors as well as clinical pharmacists and pharmacists	An educational lecture on AWaRe classification of antibiotics and the risk of AMR, in addition to a presentation of antimicrobial prescribing guidelines in the hospital.	Knowledge, perceptions, attitudes, practices (antibiotic dispensing and antibiotic prescribing)	Knowledge regarding the meaning and purpose of AWaRe classification of antibiotics increased from 39.1% to 75.4%; participants who agreed with following the AWaRe classification in their practice increased from 21.7% to 58.5%. Hospital antibiotic use of the Access group increased by 6.6% from pre- to post-intervention. The use of the Watch and Reserve group decreased by 1.7%, and 43.1%, respectively.
Afzal *et al.* (2024)^12^	Pakistan	Non-controlled before-and-after study	124 doctors, nurses, pharmacists, dispensers, and technicians	Pharmacist-led face-to-face lecture on AMS, assisted by an education guide	Knowledge, attitudes practices	After educational intervention, there was a significant improvement in the knowledge, attitude, and practice of respondents (*P*-value <0.001)
Armas Freire *et al.* (2022)^13^	Ecuador	Quasi-experimental crossover study	91 internal medicine and ICU doctors	Two educational interventions wherein one group received AMR training by face-to-face methodology and training in antimicrobial prescription practice (APP) by E-learning methodology; the other group received AMR training by E-learning methodology and APP by face-to-face methodology	Knowledge, attitudes practices (antibiotic prescribing)	Both methodologies improved knowledge, attitudes and referred practices. For the empirical treatment of pneumonia, the mean number of antibiotics was reduced from 1.87 before to 1.05 after the intervention (*P* = 0.003), whereas in the targeted management of bacteraemia, the number of antibiotics was reduced from 2.19 to 1.53 (*P* = 0.010).
Ashiru-Oredope *et al.* (2022)^14^	Multi-country	Mixed-methods	74 pharmacists, doctors, nurses, students, support staff, laboratory staff, other clinical staff, epidemiologists, academics, environmental health staff	An AMS game designed to support education on antimicrobial stewardship	Knowledge	The majority (75.7%) of respondents agreed that they got to know more about AMS after playing the game. Qualitatively, respondents reported gaining knowledge on the relevance of ‘One Health’ in AMS and others perceived the game as an opportunity to refresh the principles and strengthen their knowledge on AMS.
Barsoumian *et al.* (2018)^15^	US	Non-controlled before-and-after study	6 infectious disease (ID) fellows	A series of simulated AMS scenarios designed to train ID fellows in the synthesis of AMS interventions	Knowledge, skills and attitudes	Fellows demonstrated improvement in AMS knowledge, skills, and attitudes and plans to solve AMS challenges. Fellows performed well on knowledge-based multiple-choice questions both before and after the series. The proportion of fellows who reported delivering education on antimicrobial stewardship increased from 83% to 100% after series completion.
Bobbitt *et al.* (2023)^16^	US	Non-controlled before-and-after study	55 inpatient nurses	A mobile microlearning platform delivered to nurses on AMS principles	Knowledge, attitudes, practices	Participants’ confidence improved in key AMS activities: (1) differentiating between colonization and infection (mean difference, 0.63; *P* < 0.001), (2) identifying unnecessary urine cultures and inappropriate treatment of urinary tract infections (mean difference, 0.94; *P* < 0.001), (3) recognizing opportunities for intravenous to oral therapy conversion (mean difference, 1.07; *P* < 0.001), and (4) assessing for antibiotic-associated adverse effects (mean difference, 0.54; *P* < 0.001).
Crader (2014)^17^	US	Non-controlled before-and-after study	17 pharmacists	Basic antimicrobial education delivered through multiple methods including live continuing education programs	Knowledge	Post-tests demonstrated improvement in scores by all pharmacists. The 17 pre-test and post-test mean scores were 49.68% and 79.24%, resulting in a mean difference of 29.56% (*P* < 0.01)
Deckx *et al.* (2018)^18^	Australia	Qualitative	14 GP trainees and GP supervisors	ChAP (Changing the Antibiotic Prescribing of General Practitioners)—an educational intervention for GP trainees (and their supervisors) in order to improve evidence-based antibiotic prescribing for non-pneumonia respiratory tract infections (RTI)	Confidence and practice (prescribing behaviour)	The intervention provided new communication skills (e.g. explicitly asking about patients’ expectations, talking patients through their examination to form an overall clinical picture) and improved the early-career GPs’ confidence in not prescribing antibiotics.
Du Yan *et al.* (2021)^19^	US	A two-arm parallel randomised control trial	45 clinicians	Arm 1: Education alone and, Arm 2: Education plus feedback intervention to reduce antibiotic prescribing for upper respiratory tract infections (URI)	Practice (antibiotic prescribing for URIs)	Antibiotic prescriptions for all conditions decreased after the intervention period. For URI—15.0% to 7.8%. Bronchitis—64.0% to 32.1%. Sinusitis—87.2% to 76.8%. Pharyngitis—74.9% to 65.5%. Reduction of antibiotic prescriptions for URI and bronchitis was greater for Arm 2 compared with Arm 1 (interaction term ratio 0.60 for URI; and 0.42 for bronchitis).
Fitzpatrick *et al.* (2021)^20^	US	Non-controlled before-and-after study	42 registered nurses (RNs)	A 2-hour interactive educational AMS session consisting of a PowerPoint lecture, discussion of embedded thought questions relative to AMS and the unit culture, and role-playing scenarios	Knowledge and empowerment	The educational program resulted in a statistically significant (*P* < 0.01) increase in both AMS knowledge and empowerment level of staff RNs. Knowledge increased in the areas of preventing AMR, rates of *Clostridium difficile* and healthcare acquired infection, AMS, antimicrobial timeouts, and the nursing role in AMS. Responses to empowerment questions were more favourable after the intervention.
Flett *et al.* (2018)^21^	US	Non-controlled before-and-after study	56 gastroenterology (GI) physicians	Delivery of spaced education module to GI faculty and fellows via e-mail; hospital-wide teaching on redundant anaerobic therapy and minor changes to AMS workflow	Knowledge and practices (redundant antibiotic [metronidazole] use)	Mean knowledge scores increased significantly from 57% to 86% (*P* < 0.001). The greatest improvement was seen for questions relating to the use of double anaerobic therapy (26%–74%) and trends in bacteroides resistance (47%–90%). The rate of redundant metronidazole days decreased from 5.47 per 1000 patient-days to 2.18 per 1000 patient-days (*P* < 0.001).
Gillespie *et al.* (2013)^22^	Australia	Non-controlled before-and-after study	100 nurses	Small group or one-to-one education sessions delivered to nurses on switching from IV to oral antibiotic use	Knowledge, awareness, and practices (IV-line use)	Following education there was an increase from 14% to 42% of instances where nurses said they would question the need for intravenous antibiotics (*P* < 0.001). Three of 6 wards reduced their IV-line days, following the second wave of education.
Hale *et al.* (2017)^23^	US	Non-controlled before-and-after study	57 nurses	12-minute online module discussing risks and best practices of antibiotic use for urinary and respiratory tract infections	Knowledge, perceptions and self-confidence	Significant improvements in knowledge and perceptions were seen in 15 of the 33 indices related to assumptions regarding antibiotic use, risks, and indicators of urinary and respiratory bacterial infections. Differences between pre/post self-confidence did not change significantly following the educational module.
Heath *et al.* (2016)^24^	Not reported	Non-controlled before-and-after study	2058 nurses	An online course (six modules) focussing on infections in older adults, emphasising AMS principles in order to engage nurses in stewardship efforts	Knowledge	Average pre-test knowledge scores increased from 69.9% to 79.3% post-test. Respondents showed the greatest increase for questions focused on recognition of fever in older adults (27%), appropriate urine sample collection technique (22%), and preventing catheter-associated urinary tract infections (15%).
Hendy *et al.* (2023)^25^	Not reported	Non-controlled before-and-after study	115 nurses	An educational program consisting of three consecutive sessions delivered to nurses on AMS. The program included interactive lectures, small group discussions, and assignments to create pathways for antibiotic drug prescription and review	Knowledge, practice, perceptions	The total satisfactory knowledge improved from 14.8% at baseline to 71.3% and 68.7% during post-training and follow up, respectively, at *P* = 0.002. Positive perceptions improved from 17.4% at baseline to 72.2% post-training, and 68.7% at follow-up, *P* = 0.000. Total practice scores improved from 29.6% at baseline to 89.6% post-training, and 87.8% at follow-up, *P* = 0.000.
Hoa *et al.* (2017)^26^	Vietnam	Two-armed cluster randomized controlled trial	299 medical personnel (medical doctors, assistant medical doctors, nurses and midwives) and pharmacy personnel (pharmacists and drug-sellers)	A multi-faceted educational intervention on knowledge and reported practice regarding acute respiratory infections (ARIs) among healthcare providers (HCPs). Intervention comprised of 12 training sessions on appropriate use, case scenario management and poster distribution.	Knowledge and practice (antibiotic prescribing)	Knowledge improved in the intervention group for ARI by 28%, antibiotic use for mild ARIs by 15% and for severe ARIs by 14%. Practical competence for a mild ARI case scenario improved by 20%. Practice regarding antibiotics for mild ARIs improved by 28%.
Kaawa-Mafigiri *et al.* (2023)^27^	Uganda	Qualitative	18 health workers +125 patients and caretakers	A training and communication intervention to improve adherence to antibiotic prescription instructions among health workers and patients	Perspectives on prescription adherence	Health workers reported improvement in adherence by patients demonstrated through changes in practices e.g. being able to describe how and when to take their prescriptions. Health workers also reported that patients expressed their appreciation for the need to first undertake tests before getting medication.
Kandeel *et al.* (2019)^28^	Egypt	Non-controlled before-and-after study	289 physicians. 596 pharmacists and 607 patients	A multifaceted intervention (including training and a communication campaign) aimed at physicians, pharmacists and patients to raise awareness of rational antibiotic prescribing for acute respiratory infections (ARIs)	Knowledge, attitudes, beliefs and practices (prescribing behaviour for ARIs)	Post-intervention, there was 23.1% decrease in antibiotic prescribing for ARIs (83.7% to 64.4%) and physicians and pharmacists self-reported less frequently prescribing antibiotics for ARIs. There was also an increase in correct responses to the clinical scenarios and in attitude and belief scores for physicians, pharmacists, and patients regarding antibiotic use.
Kolman *et al.* (2016)^29^	South Africa	Non-controlled before-and-after study	10 pharmacy personnel (five pharmacists, two pharmacist interns and three pharmacist’s assistants)	A comic book application (app) and educational session to engage pharmacy personnel in AMS, and improve their knowledge of infectious diseases	Knowledge and attitudes towards AMS and infectious diseases	Infectious disease knowledge of AMS improved from 66% versus 96% for the pre- and post-tests, respectively (*P* < 0.05). The largest percentage change pertained to question 4 on antibiotic drug of choice for ESBL producing *K. pneumoniae* (from 40% to 100%) and question 5 on identifying antibiotics with anaerobic coverage (from 40% to 100%).
Kpokiri *et al.* (2022)^30^	Ghana	Mixed-methods	68 health professionals including pharmacists, physicians, nurses, laboratory and biomedical scientists	3-day training session on AMR and AMS quality improvement	Knowledge, awareness and practices	The majority of participants confirmed that the training was very helpful and increased their understanding of AMR. Participants self-reported changes in their practice, such as reduced empirical prescription of antibiotics and more detailed counselling for patients on antibiotic use. Participants felt more confident carrying out AMS roles after the training.
Lee and Stead (2021)^31^	US	Non-controlled before-and-after study	10 advanced practice practitioners (APPs) from cardiac surgery and neurocritical care	An educational intervention consisting of a 20-to-25-minute in-person interactive learning session using a case-based format to review the diagnosis and management of asymptomatic bacteriuria (ASB) and candiduria (ASC)	Knowledge and practice (prescribing behaviours)	There was improvement in knowledge from pre-intervention (2.57 mean score to post-intervention (mean score 4.00, *P* < 0.05). There was a decrease in urine cultures sent by APPs between the pre-intervention (90 urine cultures) and post-intervention (63 urine cultures) periods.
Leppien *et al.* (2019)^32^	US	Non-controlled before-and-after study	58 healthcare clinicians (providers, pharmacists and nurses)	An education intervention to improve knowledge and antibiotic prescribing for suspected UTI involving either a live lecture or independent study	Knowledge and practice (prescribing behaviours)	Knowledge: Post-test scores among those who received live educational training were significantly higher (*P* < 0.001) than the post-test scores for those who independently reviewed educational materials. Prescribing trends: the number of urine samples collected for suspected UTI decreased from 48 to 11. This coincided with a decreased rate of inappropriate antibiotic treatment from nine to zero percent.
Lutfiyati *et al.* (2023)^33^	Indonesia	Non-controlled before-and-after study	36 health professionals (including medical doctors, pharmacists, nurses, laboratory analysts)	2-day educational workshop on AMS over Zoom including PowerPoint presentations and case scenarios	Knowledge and attitudes	The level of knowledge of health professionals before and after the workshop had no significant changes because knowledge levels were already high pre-workshop. However, their perception (attitudes) increased significantly after the educational intervention.
Magin *et al.* (2016)^34^	Australia	Non-controlled before-and-after study	75 GP Registrars	A 90-min face-to-face workshop supported by online modules	Intentions to prescribe	There were statistically significant reductions in intentions to prescribe antibiotics for sore throat (24.0% absolute reduction), otitis media (17.5% absolute reduction) and two of three acute bronchitis (12.0% and 18.0% absolute reduction) vignettes
Manning *et al.* (2022)^35^	US	Non-controlled before-and-after study	47 nurses from the surgical intensive care unit (SICU) and bone marrow transplant unit (BMTU)	A training program designed to equip nurses with nursing-specific knowledge and skills in AMS, comprised of four, online, asynchronous, sequential, self-paced learning modules covering AMS topics	Knowledge, perceptions, confidence	There was a statistically significant increase in participant perceptions that conducting patient antibiotic allergy assessments, reviewing preliminary microbiology culture results to determine antibiotic appropriateness, assuring antibiotic duration and indications are recorded in the electronic medical record, and transitioning antibiotics from intravenous to oral contribute to AMS processes (*P* < 0.05).
McNulty *et al.* (2018)^36^	UK	Randomized controlled trial	166 GPs, 51 nurses and 101 other staff including receptionists, healthcare assistants and practice managers	A TARGET antibiotic interactive workshop designed to improve antibiotic dispensing in general practice including a presentation, reflection on antibiotic data, promotion of patient and GP staff resources, clinical scenarios and action planning.	Practices (antibiotic dispensing)	An intention-to-treat analysis revealed that total antibiotic dispensing was 2.7% lower in intervention practices (*P* = 0.06) compared with controls. Dispensing in intervention practices was 4.4% lower for amoxicillin/ampicillin (*P* = 0.02); 5.6% lower for trimethoprim (*P* = 0.03); and a non-significant 7.1% higher for nitrofurantoin (*P* = 0.06).
Medina-Presentado *et al.* (2017)^37^	Multi-country	Non-controlled before-and-after study	535 physicians, nurses and microbiologists	A six-week online continuing interprofessional interactive educational program on hospital-acquired infections and AMR for Latin America including: Reading resources, videos and voice-over-presentations, patient aids, and electronic rounds (e-rounds) on clinical cases and clinical simulations.	Knowledge	There was a significant increase in knowledge between before and after the course. Of 535 participants who took both tests, the grade increased from 59% to 81% (*P* < 0.001).
Menon *et al.* (2020)^38^	Australia	2-arm, non-controlled before-and-after study	63 Junior doctors (*n* = 29 for online intervention and *n* = 34 for face-to-face FTF)	2-Arm before-and-after study evaluating the impact of online (via Qstream) spaced education versus FTF tutorials for improving knowledge and confidence of junior doctors regarding antimicrobial prescribing	Knowledge and confidence	The Qstream group mean knowledge score increased from 69.7% to 81.5% (*P* = 0.003). The FTF group mean knowledge score increased from 67.6% to 67.9% (*P* = 0.92). The improvement in knowledge was greater in the Qstream cohort compared with the FTF cohort (*P* = 0.01). The Qstream group also improved their mean self-reported confidence from 5.14 to 6.55 (*P* < 0.001). The FTF group improvement from 5.37 to 5.85 was not significant (*P* = 0.06).
Michener *et al.* (2018)^39^	US	Non-controlled before-and-after study	Between 68–108 physicians, nurse practitioners, physician assistants, pharmacists, and nurses caring for older adults in outpatient, long-term, and acute care settings	Five topic case-based discussion series with the goal of improving knowledge related to the recognition, diagnosis, and management of infections common among older adults	Knowledge	The average pre-course score was 3.6/5 (72%) compared to 3.9/5 (78%, *P* = 0.06) post-course, indicating improved knowledge. All respondents indicated they were somewhat or highly likely to implement change. Assessed using a 3-point scale, the average score ranged from 2.50 for upper respiratory tract infections to 2.89 for skin and soft tissue infections.
Mittal *et al.* (2023)^40^	India	Non-controlled before-and-after study	190 nurses in inpatient and intensive care units	An educational program comprised of four modules designed to increase awareness on AMR, AMS and antimicrobial use among nurses	Knowledge, attitudes and beliefs towards antibiotics, antibiotic use and resistance	Significant improvement in the post-program knowledge scores pertaining to all the learning modules was seen compared to the pre-program scores. Similarly, there was improvement in the number of correct responses for items pertaining to module 2 (infection prevention and control) like healthcare associated infections, culture techniques and biomedical waste management.
Narayanan *et al.* (2019)^41^	US	Non-controlled before-and-after study	Sample size not reported. Healthcare staff including medical residents, nurse practitioners, physician assistants and staff physicians from 3 internal medicine (IM) wards	Two interventions which included: 1) information technology (IT) intervention which involved redesign of urine culture (UC) ordering and 2) an educational intervention which involved active and passive education of prescribers specifically designed to address unnecessary treatment of asymptomatic bacteriuria (ASB)	Practices (treatment practices for ASB and UC ordering)	There was not a reduction in inappropriate treatment of ASB compared to baseline after the IT intervention (48% versus 42%). Following the education intervention, there was a reduction in unnecessary ASB treatment compared both to baseline (35% versus 42%) and to post-IT intervention (35% versus 48%). There was no difference in the percentage of total UCs ordered by clinicians.
Ng *et al.* (2021)^42^	UK	Non-controlled before-and-after study	28 pharmacists	A workshop and workplace-based improvement project aimed to facilitate learner capability, opportunity, and motivation to lead AMS activities in the workplace	Knowledge and practice (implementation of improvement projects)	Mean knowledge scores increased from 67.7% pre- intervention to 81.1% post-intervention (*P* < 0.0001). Of the 21 responses to a follow-up survey, ongoing quality improvement work and improvement outcomes were reported by nine and six learners, respectively. Improvement projects included prospective audit and feedback, use of practice aids and education and promotion.
Nyamu *et al.* (2021)^43^	Kenya	Non-controlled before-and-after study	Not reported—family medicine residents, supervising faculty and community health volunteers (CHVs)	A participatory, interactive educational intervention with health facility staff and CHVs to highlight the burden of upper respiratory tract infections (URTIs) as the most common presenting ailment in the health facilities, and to increase adherence to treatment guidelines.	Practice (antimicrobial prescription)	Antimicrobial prescription in under 5 s group was reduced by 44% in the 2 weeks following the intervention, and by 18% at weeks 8–9. In over 5 s, prescribing was reduced by 18% and 8%, respectively.
Parzen-Johnson *et al.* (2023)^44^	US	Non-controlled before-and-after study	Advanced Practice Practitioners (nurse practitioners and physician’s assistants)	An antibiotic stewardship education curriculum targeted to APPs. The course was a combination of lecture followed by problem-based learning (PBL).	Subjective opinions and knowledge on AMS and practice (prescriptions)	Subjective opinions and comfort with basic AMS principles increased from pre-intervention. Correct responses to knowledge-based assessments increased from baseline and were maintained at 6-month follow-up (*P* = 0.03). Simple skin and soft tissue infection prescriptions for clindamycin went from 44.4% pre-intervention to 26.5% (*P* = 0.2) post-intervention.
Pisano *et al.* (2016)^45^	N/A (online)	Non-controlled before-and-after study	39 internal medicine residents (IMRs)	Use of social media (Facebook and Twitter) to increase IMRs antibiotic knowledge and awareness of the study antibiotic stewardship program (ASP)	Social media use, knowledge, attitudes and beliefs regarding antimicrobial use and antibiotic resistance, and awareness of the ASP and its resources	Knowledge-based test scores improved (12 versus 13; *P* = 0.048) as a result of the intervention. Increased knowledge scores in residents that interacted with the platforms were seen but did not reach significance. Participants who completed an infectious diseases (ID) consult rotation during the intervention time period did not have significantly better scores on the knowledge-based portion of the survey, 10.55 (no ID consult rotation) versus 11.94 (ID consult rotation).
Saleh *et al.* (2021)^46^	Jordan	Non-controlled before-and-after study	100 community pharmacists	An educational workshop conducted on Zoom over two days to improve knowledge and perceptions of community pharmacists on AMS and enhance their ability to appropriately select correct antibiotic therapy. Each session was an hour and used PowerPoint presentations, videos, and clinical case scenarios	Awareness and perception on AMS and practice (ability to select correct antibiotic therapy)	Knowledge scores improved following the educational workshop with a median score of 7/10, *P* < 0.001. Knowledge of the definition AMS increased from one third pre-workshop to 56% post- workshop. Pharmacists’ ability to select correct antibiotic therapy significantly improved post- workshop (*P* < 0.05). Perceptions towards AMS were consistently positive before and after the workshop.
Schwartz *et al.* (2017)^47^	US	Randomised control trial	Not reported, 80% of resident and 50% of attending physicians	Two interventions—one physician education (12 sessions) and the other audit and feedback deployed to reduce antimicrobial errors and use.	Practices (antimicrobial errors and use)	Neither intervention (physician education or audit and feedback) was associated with reductions in antimicrobial use or error rates compared with the control.
Shane (2021)^48^	US	Non-controlled before-and-after study	10 nurse practitioners (NPs)	An eLearning module designed to educate NPs in long term care facilities (LTCF) about AMS specific to suspected UTIs	Knowledge	A significant difference was observed between the number of correct answers on the pre-test (mean 6.4) and the post-test (mean = 8.1, *P* = 0.006), suggesting the eLearning module increased knowledge of NP participants in an LTCF setting.
Sharp *et al.* (2017)^49^	US	Stepped-Wedge Cluster Randomised Trial	105 primary care and 21 urgent care outpatient clinics	Clinical Decision Support + provider education. The education included a recorded online presentation and 2 webinars delivered in the middle of the staggered rollout.	Practice (antibiotic prescribing)	Provider education showed significant decreases in antibiotic prescribing (aOR, 0.51; 95% CI, 0.46–0.57), but these effects were not sustained over the study period
Sneddon *et al.* (2018)^50^	Multi-country	Post-hoc survey	409 healthcare professionals including pharmacists, clinicians, nurses, microbiologists)	An online interactive course spanning 6 weeks on AMS and AMR. Materials included articles to read, videos to watch, short audio pieces, case studies, discussion steps, quizzes, and submission of written pieces for peer review.	Practice (implementation of AMS interventions)	160/409 participants who completed an implementation survey 6 months after course completion reported that they had implemented stewardship interventions after completing the course.
Sneddon *et al.* (2020)^51^	Ghana	Non-controlled before-and-after study	51 health professionals including nurses, medical doctors, pharmacists, laboratory scientists, hospital managers, midwives and a public health practitioner	A training session held across two hospitals, comprised of a mix of lectures and interactive case/scenario-based sessions to engage participants in discussion of current practice and explore behaviours and attitudes to using antibiotics	Knowledge, attitudes, practices (use of antibiotics)	Comparison of pre- and post-test scores (Hospital A: 9.4 and 10.9, Hospital B: 9.2 and 11.1, respectively) demonstrated statistically significant improvement in knowledge of antimicrobial resistance and appropriate use of antibiotics. Participants from both hospitals demonstrated improved attitudes and behaviours around the use of antibiotics after the training session.
Tahoon *et al.* (2020)^52^	Egypt	Non-controlled before-and-after study	48 health professionals including medical doctors, nurses and pharmacists	Educational sessions comprised of lectures, clinical scenarios, guidelines and a Whatsapp group designed to educate healthcare professionals about AMS	Knowledge, attitudes and practices	Knowledge increased among physician and pharmacists from 39.3% to 100% (*P* < 0.001) and among nurses from 13.3/20 to 18.0/20 (*P* < 0.001). Attitudes among physicians and pharmacists increased from 85.7% to 100% (*P* < 0.05) and among nurses from 14.8/16 to 15.7/16 (*P* < 0.001). Physicians’ good practice increased from 31.8% to 95.5% and also for nurses from 9.3/14 to 11.5/14. There was no significant improvement in practice of pharmacists (*P* > 0.05).
Velez *et al.* (2014)^53^	US	Non-controlled before-and-after study	18 Medical doctors (MDs), nurse practitioners, (NPs) and physician assistants (PAs)	Online education on community-acquired methicillin-resistant *Staphylococcus aureus* (CA-MRSA) delivered via noninteractive voice over PowerPoint (VOPP) to prescribers	Knowledge, Practices (prescribing behaviours) and guideline adherence	12 of the 18 participants showed an increase in knowledge. Forty-three per cent of prescribers’ charts demonstrated improved practice through use of the guidelines. Out of 18 prescribers, 44% sent a total of 21 cultures for abscesses. There was no difference in practice behaviours between professional groups.
von Schreeb *et al.* (2020)^54^	Macedonia	Non-controlled before-and-after study	38 physicians from paediatrics, internal medicine, gynaecology and obstetrics, general practice and family medicine, surgery, urology, infectious diseases, medical microbiology	A MOOC designed to improve knowledge on AMS	Knowledge and intention to change behaviour	Mean course scores increased from 77.8% to 82.2%, *P* = 0.007. The highest improvement was seen in the category ‘managing infections’ in which participants’ knowledge increased by 10.2%. Seventy-five percent of respondents deemed themselves likely or very likely to change their behaviour. Seven percent did not expect to modify their clinical practice.
Yamamoto *et al.* (2018)^55^	Japan	Non-controlled before-and-after study	38 physicians	A course consisting of lectures using illness scripts and checklists, interactive communication skills training using role-playing, and vignette-based evaluation to improve clinician skills in the daily clinical practice of treating acute respiratory tract infections (RTIs)	Knowledge and attitudes	There were reductions in the intention to prescribe antibiotics for 4/6 non-pneumonia RTI vignettes: acute bronchitis (47.2% to 28.1%), common cold (16.2% to 1.6%), acute pharyngitis (27.0% to 5.0%), and acute rhinosinusitis (33.3% to 13.3%).
Yasein *et al.* (2021)^56^	Jordan	Randomised control trial	69 resident physicians from internal medicine, pediatrics, emergency, Ear, Nose, and Throat (ENT), and family medicine department	An educational intervention that was implemented over a period of two weeks and consisted of two educational seminars covering topics related to the prescription of antimicrobial agents and resistance against them	Knowledge, attitudes and behaviour intention	Post-intervention, the rate of antimicrobial prescribing in the intervention group was higher than that of the control group (*P* < 0.001). Mean ‘fear’ score for the intervention group was lower than that for the control group post-intervention, *P* = 0.027. Mean scores for attitude, behavioral intention, and knowledge dimensions did not change post-intervention significantly. Results are highly likely to have been impacted by the COVID-19 outbreak.

### Data analysis

Individual study characteristics and findings were summarised, and similarities, differences and patterns identified. To identify discernible patterns of effectiveness, all studies were mapped across five categories of intervention effectiveness, following Price *et al*.^57^ These categories were based upon both the strength of the evidence and whether the primary outcome could evidence actual behaviour change or its antecedents (i.e. knowledge, attitudes, perceptions). The five categories of a relative measure of effectiveness included: (i) interventions indicative of clear positive behaviour change in the desired direction; (ii) interventions indicative of some positive behaviour change in the desired direction; (iii) interventions indicative of a positive effect on the antecedent of behaviour, such as knowledge or awareness, in the desired direction; (iv) interventions indicative of no effect on behaviour or antecedents of behaviour; and (v) interventions indicative of a negative effect on behaviour or antecedents of behaviour in a non-desired direction.

## Results

**Figure 1.**
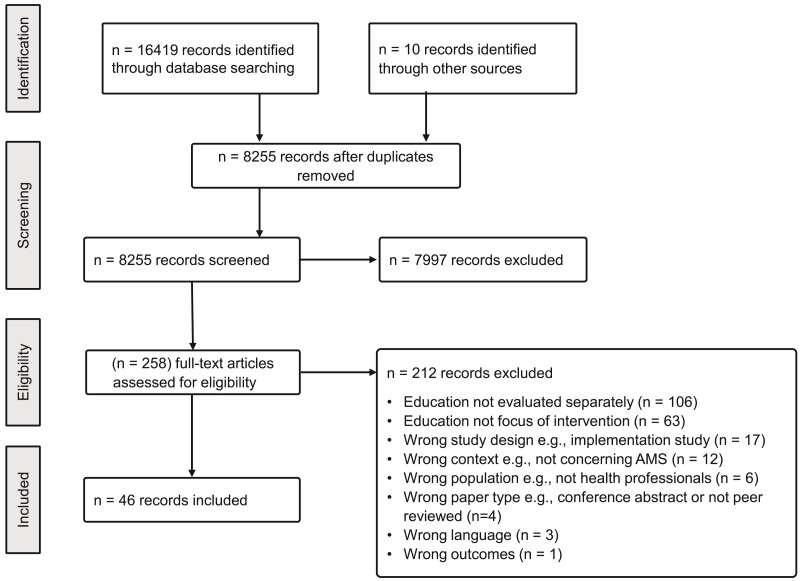
PRISMA flowchart.

The initial searches were completed in December 2023, with an additional search conducted in July 2024. Database searches of MEDLINE, SCOPUS, Embase, CINAHL, Web of Science, CENTRAL and PsychINFO yielded a total of 8255 records. A further ten were identified through manual references searches of the included papers. A total of 258 full text studies were assessed for eligibility, of which 212 were excluded. Articles were excluded for the following reasons: the educational component was not evaluated separately (*n* = 109); education was not the focus of intervention (*n* = 63); wrong study design e.g. implementation study (*n* = 17); wrong context e.g. not concerning AMS (*n* = 12); wrong population e.g. not health professionals (*n* = 6); wrong paper type e.g. conference abstract or not peer reviewed (*n* = 4); paper not available in English (*n* = 3); wrong outcomes (*n* = 1). Following screening, 46 studies that matched the eligibility criteria were included in the review. A detailed process of study selection is presented in Figure 1.

### Study characteristics

#### Study design

The study designs of the 46 records for review include RCTs (*n* = 5), a stepped-wedge cluster randomised trial (*n* = 1) and non-controlled before-and-after studies (*n* = 40). Of the latter non-controlled studies, 37 were quantitative non-randomised studies; two were qualitative studies; and one a mixed-methods study.

#### Country

Of the 46 studies, approximately half were conducted in high income countries (*n* = 24). Of this 24, 17 were conducted in the USA, and the rest in Australia, Japan and the UK. The remaining were conducted in low- to middle-income countries (*n* = 16) or in multiple countries (*n* = 3). Two studies did not report the country, and one was conducted online.

#### Type of education intervention

The most common types of interventions were education sessions delivered to a cadre of health professional. The mode and type of education delivered was heterogenous, ranging from single lectures to courses spanning several weeks, some online and others face-to-face. Education sessions were often multi-modal, with formal education alongside a wide variety of other educational materials and resources. There were interventions that focused on AMS in general, while others targeted a specific learning objective e.g. aiming to reduce antibiotic prescriptions for upper respiratory tract infections. Overall, there was no consistency in what constituted an ‘educational intervention’, with little reuse of materials, and almost no consideration of psychological or pedagogical theory or practice in intervention design.

Comparators consisted of either practice as normal^36,56^ or an alternate intervention^19,26^ or both.^47^ One study used a stepped wedge design to account for confounders.^49^ Although included in the review, Du Yan *et al*.’s^19^ study is problematic in that education alone was compared to education with audit and feedback, such that the control for education was an improved intervention. It should be understood as a compromised design in the context of this specific review of the effectiveness of post-professional education for AMS.

#### Target audience

The target audience varied, with some focused solely on doctors or nurses while others sought to target a range of health professionals at once, including doctors, nurses, pharmacists, and laboratory professionals. Those focused on behaviour change were targeted at prescribers only, the profession of which varies somewhat across countries, being mostly doctors but also senior nurses and pharmacists in some circumstances. Sixteen studies focused on multiple professions, 13 on doctors only, 9 on nurses only, 4 pharmacists only, 2 doctors and pharmacists, and 2 nurses and assistant doctors. Most studies focused on effecting behaviour change—i.e. prescribing practice—were targeted at doctors. Studies focused on changing antecedents of behaviour—i.e. knowledge and attitudes—were more often, though not exclusively, focused on nurses, pharmacists and multi-disciplinary teams. This work tended to be lower quality non-controlled before-and-after educational interventions.

#### Outcome measures

The most common outcome measures were changes in knowledge, attitudes, and intentions to change practice (*n* = 24). Almost half of the total studies also focused on changing behaviour (*n* = 22). Studies focused on behaviour change generally also included measures of change in knowledge and/or attitudes. Behaviour change measures primarily focused on prescribing practice, measured as reductions in antibiotic prescribing, changed antibiotic prescribing or assessment of antibiotic prescribing error.

### Quality of studies

#### EPOC

Among the included studies, six met the EPOC study design criteria (see Table 3). Two studies had low risk of bias across all indicators.^19,36^ Schwartz *et al*.^47^ and Yasein *et al*.^56^ scored high risk of bias for random sequence generation and baseline characteristics, respectively. Hoa *et al*.,^26^ Schwartz *et al*.^47^ and Yasein *et al*.^56^ had incomplete information for several indicators. Sharp *et al*.^49^ was a stepped-wedge cluster randomised trial and was assessed using interrupted time series design criteria, and scored ‘low’ for risk of bias across all relevant EPOC categories.

**Table 3.** Risk of bias studies meeting the EPOC criteria

Study	Random sequence generation	Allocation concealment	Baseline outcome measurements similar	Baseline characteristics similar	Incomplete outcome data	Knowledge of the allocated interventions adequately prevented during the study	Protection against contamination	Selective outcome reporting	Intervention independent of other changes	Shape of the intervention effect pre-specified	Intervention unlikely to affect data collection	Other risks of bias
Du Yan *et al.* (2021)^19^	L	L	L	L	L	L	L	L	N/A	N/A	N/A	L
Hoa *et al.* (2017)^26^	L	L	L	U	U	U	U	L	N/A	N/A	N/A	U
McNulty *et al.* (2018)^36^	L	L	L	L	L	L	L	L	N/A	N/A	N/A	L
Schwartz *et al.* (2017)^47^	H	L	L	U	L	U	U	L	N/A	N/A	N/A	U
Sharp *et al.* (2017)^49^	N/A	N/A	N/A	N/A	L	L	N/A	L	L	L	L	L
Yasein *et al.* (2021)^56^	L	L	L	H	L	U	U	L	N/A	N/A	N/A	U

Categories specific to interrupted time series (ITS) design were excluded as they were not included in the review (i.e. ‘intervention independent of other changes’, ‘shape of the intervention effect pre-specified’ and ‘intervention unlikely to affect data collection’).

H, high risk of bias; L, low risk of bias; U, unclear risk of bias.

#### MMAT

Studies that were assessed as per the MMAT criteria (*n* = 40) included: quantitative non-randomised studies (*n* = 37); qualitative studies (*n* = 2); and mixed-methods studies (*n* = 1). A summary of the MMAT appraisal tool for the 37 studies can be seen in Table 4. Only two studies scored the maximum 5* overall quality rating, both qualitative.^18,27^ Six were scored 4*, all of which failed to account for confounders in the design or analysis.^13,17,20,28,40,52^ All others were scored 3* (*n* = 25) or 2* (*n* = 7) (60% or lower quality criteria met). Only those studies that scored 4* or above in the MMAT overall ratings (80% or greater quality criteria met) will be considered further with regard to effectiveness (*n* = 8), alongside those that met the criteria for EPOC risk of bias assessment (*n* = 6), albeit with considerable caution given the failure of these studies to account for confounding variables.

**Table 4.** Mixed Methods Appraisal Tool (MMAT) Assessment

Quantitative non-randomized								
Study	Are there clear research questions?	Do the collected data allow to address the research questions?	Are the participants representative of the target population?	Are measurements appropriate regarding both the outcome and intervention (or exposure)?	Are there complete outcome data?	Are the confounders accounted for in the design and analysis?	During the study period, is the intervention administered (or exposure occurred) as intended?	Overall Quality Rating
Abu-Ajaleh *et al.* (2023)^11^	Y	Y	CT	Y	Y	N	Y	***
Afzal *et al.* (2024)^12^	Y	Y	CT	Y	Y	N	Y	***
Armas Freire *et al.* (2022)^13^	Y	Y	Y	Y	Y	N	Y	****
Ashiru-Oredope *et al.* (2022)^14^	Y	Y	CT	Y	Y	N	Y	***
Barsoumian *et al.* (2018)^15^	Y	Y	CT	Y	Y	N	Y	***
Bobbitt *et al.* (2023)^16^	Y	Y	CT	Y	Y	N	Y	***
Crader (2014)^17^	Y	Y	Y	Y	Y	N	Y	****
Fitzpatrick *et al.* (2021)^20^	Y	Y	Y	Y	Y	N	Y	****
Flett *et al.* (2018)^21^	Y	Y	CT	Y	Y	N	Y	***
Gillespie *et al.* (2013)^22^	Y	Y	CT	Y	Y	N	Y	***
Hale *et al.* (2017)^23^	Y	Y	CT	Y	Y	N	Y	***
Heath *et al.* (2016)^24^	Y	Y	CT	Y	Y	N	Y	***
Hendy *et al.* (2023)^25^	Y	Y	CT	Y	Y	N	Y	***
Kandeel *et al.* (2019)^28^	Y	Y	Y	Y	Y	N	Y	****
Kolman *et al.* (2016)^29^	Y	Y	CT	Y	Y	N	Y	***
Kpokiri *et al.* (2022)^30,a^	Y	Y	CT	Y	Y	N	Y	**
Lee and Stead (2021)^31^	Y	Y	CT	Y	Y	N	Y	***
Leppien *et al.* (2019)^32^	Y	Y	CT	Y	Y	N	Y	***
Lutfiyati *et al.* (2023)^33^	Y	Y	CT	Y	Y	N	Y	***
Magin *et al.* (2016)^34^	Y	Y	CT	Y	Y	N	Y	***
Manning *et al.* (2022)^35^	Y	Y	CT	Y	Y	N	Y	***
Medina-Presentado *et al.* (2017)^37^	Y	Y	CT	Y	Y	N	Y	***
Menon *et al.* (2020)^38^	Y	Y	CT	Y	Y	N	Y	***
Michener *et al.* (2018)^39^	Y	Y	CT	Y	Y	N	Y	***
Mittal *et al.* (2023)^40^	Y	Y	Y	Y	Y	N	Y	****
Narayanan *et al.* (2019)^41^	Y	Y	CT	Y	Y	N	Y	***
Ng *et al.* (2021)^42^	Y	Y	CT	Y	Y	N	Y	***
Nyamu *et al.* (2021)^43^	Y	Y	CT	Y	Y	N	Y	***
Parzen-Johnson *et al.* (2023)^44^	Y	Y	CT	Y	Y	N	Y	***
Pisano *et al.* (2016)^45^	Y	Y	CT	Y	Y	N	Y	***
Saleh *et al.* (2021)^46^	Y	Y	CT	Y	Y	N	Y	***
Shane (2021)^48^	Y	Y	N	Y	CT	N	Y	**
Sneddon *et al.* (2018)^50^	Y	Y	CT	Y	CT	N	Y	**
Sneddon *et al.* (2020)^51^	Y	Y	CT	Y	CT	N	Y	**
Tahoon *et al.* (2020)^52^	Y	Y	Y	Y	Y	N	Y	****
Velez *et al.* (2014)^53^	Y	Y	CT	Y	Y	N	Y	***
von Schreeb *et al.* (2020)^54^	Y	Y	CT	Y	CT	N	Y	**
Yamamoto *et al.* (2018)^55^	Y	Y	CT	Y	Y	N	Y	***
**Qualitative**								
Study	Are there clear research questions?	Do the collected data allow to address the research questions?	Is the qualitative approach appropriate to answer the research question?	Are the qualitative data collection methods adequate to address the research question?	Are the findings adequately derived from the data	Is the interpretation of results sufficiently substantiated by data?	Is there coherence between qualitative data sources, collection, analysis and interpretation?	Overall Quality Rating
Deckx *et al.* (2018)^18^	Y	Y	Y	Y	Y	Y	Y	*****
Kaawa-Mafigiri *et al.* (2023)^27^	Y	Y	Y	Y	Y	Y	Y	*****
Kpokiri *et al.* (2022)^30,^a^^	Y	Y	Y	Y	CT	N	Y	**
**Mixed-methods**								
Study	Are there clear research questions?	Do the collected data allow to address the research questions?	Is there an adequate rationale for using a mixed methods design to address the research question?	Are the different components of the study effectively integrated to answer the research question?	Are the outputs of the integration of qualitative and quantitative components adequately interpreted?	Are divergences and inconsistencies between quantitative and qualitative results adequately addressed?	Do the different components of the study adhere to the quality criteria of each tradition of the methods involved?	Overall Quality Rating
Kpokiri *et al.* (2022)^30,a^	Y	Y	CT	Y	Y	CT	N	**

Overall quality is assessed on the basis of how many of the quality criteria are met, with 5* being the maximum and 1* the minimum (the first two quality assessment items, consistent across all sets of criteria, in the MMAT are not included in this scoring as they are the baseline for inclusion).

Y, Yes; N, No; CT, Cannot Tell.

^a^Kpokiri *et al*. (2022) is a mixed-methods study and so assessed against all three sets of criteria, with overall quality assessed as the score of the weakest component of the three sets of criteria.

### Relative effectiveness of interventions

Reviewed interventions were grouped into five categories of relative measures of effectiveness. As shown in Table 5, two studies demonstrated a clear desired behaviour change following the intervention,^19,36^ while eleven studies resulted in some desired behaviour change.^11,13,15,21,28,32,41,42,43,44,49^ A positive effect on the antecedent of behaviour was reported in 30 papers. Three studies showed no effect on behaviour change or antecedents of behaviour change.^14,47,56^ Overall, forty-three studies demonstrated some positive effect on the outcomes of interest i.e. improved knowledge, awareness, attitudes, practices, and behaviours around AMR and AMS, albeit all but 6 of these studies were non-controlled with a high risk of bias.

**Table 5.** Patterning of effectiveness across the type of target population

Study	Clear positive behaviour change	Some positive behaviour change	Positive effect on the antecedent of behaviour	No effect on behaviour or antecedents of behaviour	Negative effect on behaviour or antecedents of behaviour
Abu-Ajaleh *et al.* (2023)^11^		Doctors and Pharmacists			
Afzal *et al.* (2024)^12^			Multi-professional		
Armas Freire *et al.* (2022)^13^		Doctors			
Ashiru-Oredope *et al.* (2022)^14^				Multi-professional^a^	
Barsoumian *et al.* (2018)^15^		Doctors			
Bobbitt *et al.* (2023)^16^			Nurses		
Crader (2014)^17^			Pharmacists		
Deckx *et al.* (2018)^18^			Doctors		
Du Yan *et al.* (2021)^19^	Doctors				
Fitzpatrick *et al.* (2021)^20^			Nurses		
Flett *et al.* (2018)^21^		Doctors			
Gillespie *et al.* (2013)^22^			Nurses		
Hale *et al.* (2017)^23^			Nurses		
Heath *et al.* (2016)^24^			Nurses		
Hendy *et al.* (2023)^25^			Nurses		
Hoa *et al.* (2017)^26^			Multi-professional		
Kaawa-Mafigiri *et al.* (2023)^27^			Multi-professional^b^ and patients		
Kandeel *et al.* (2019)^28^		Doctors and Pharmacists			
Kolman *et al.* (2016)^29^			Pharmacists		
Kpokiri *et al.* (2022)^30^			Multi-professional		
Lee and Stead (2021)^31^			Nurses and (assistant) Doctors		
Leppien *et al.* (2019)^32^		Multi-professional			
Lutfiyati *et al.* (2023)^33^			Multi-professional		
Magin *et al.* (2016)^34^			Doctors		
Manning *et al.* (2022)^35^			Nurses		
McNulty *et al.* (2018)^36^	Doctors				
Medina-Presentado *et al.* (2017)^37^			Multi-professional		
Menon *et al.* (2020)^38^			Doctors		
Michener *et al.* (2018)^39^			Multi-professional		
Mittal *et al.* (2023)^40^			Nurses		
Narayanan *et al.* (2019)^41^		Multi-professional			
Ng *et al.* (2021)^42^		Pharmacists			
Nyamu *et al.* (2021)^43^		Multi-professional^b^			
Parzen-Johnson *et al.* (2023)^44^		Nurses and (assistant) Doctors			
Pisano *et al.* (2016)^45^			Doctors		
Saleh *et al.* (2021)^46^			Pharmacists		
Schwartz *et al.* (2017)^47^				Doctors	
Shane (2021)^48^			Nurses		
Sharp *et al.* (2017)^49^		Multi-professional^b^			
Sneddon *et al.* (2018)^50^			Multi-professional^b^		
Sneddon *et al.* (2020)^51^			Multi-professional		
Tahoon *et al.* (2020)^52^			Multi-professional		
Velez *et al.* (2014)^53^			Multi-professional		
von Schreeb *et al.* (2020)^54^			Doctors		
Yamamoto *et al.* (2018)^55^			Doctors		
Yasein *et al.* (2021)^56^				Doctors	

^a^Multi-professional usually involves doctors, nurses and pharmacists (occasionally lab technicians).

^b^Unclear about specific profession of those receiving intervention.

Of the 14 studies included for analysis of effectiveness on the basis of adequate quality, however, we found two studies with educational interventions demonstrating clear behaviour change,^19,36^ three studies providing some evidence for behaviour change,^13,28,49^ seven studies reporting a positive effect on the antecedents of behaviour change,^17,18,20,26,27,40,52^ and two studies which found no effect on behaviour or the antecedents of behaviour, as detailed below.^47,56^

#### Clear behaviour change

Two of the three studies with a low risk of bias reported a clear indication of behaviour change in the desired direction.^19,36^ However, Du Yan *et al*.^19^ was not properly controlled in the terms of this review given our focus on the efficacy of education alone, as the education only arm was the control group in this study, so the findings must be treated with caution.

Forty-five clinicians in the Du Yan *et al*.^19^ study took part in a 2-arm parallel group RCT to address acute respiratory infections (ARIs). The control group received education alone (treatment guidelines, presentation and online course) with the intervention group receiving the same education plus individualised feedback via an online dashboard. Reductions in antibiotic prescribing rates occurred across both arms after the intervention. Prescribing for upper respiratory tract infections reduced from: 18.4 pre to 12.8% post; bronchitis—46.8 pre to 35.3% post; sinusitis—84.1 pre to 76.7% post and pharyngitis—81.3 pre to 75.3% post in the education only arm. The study showed that reduction of antibiotic prescriptions for URI and bronchitis was greater for the group receiving the education intervention plus individualized feedback compared with that for the group receiving the education intervention alone, and there was some evidence that the learning effect while large did not persist over time.

The McNulty *et al*.^36^ study consisted of a RCT to determine the impact of a TARGET workshop on antibiotic dispensing across 36 GP practices in the UK. The workshop involved a presentation, reflection on antibiotic data, promotion of patient and general practice staff resources, clinical scenarios and action planning. Seventy-five practices were the control (usual practice). In an intention-to-treat analysis, total antibiotic dispensing was 2.7% lower in intervention practices (95% CI −5.5% to 1%, *P* = 0.06) compared with controls. The Complier Average Causal Effect analysis, which estimates impact in those that comply with assigned intervention, indicated 6.1% (95% CI 0.2%–11.7%, *P* = 0.04) lower total antibiotic dispensing in intervention practices.

#### Some behaviour change

The controlled study by Sharp *et al*.^49^ had a low risk of bias and reported evidence of some positive behaviour change in the desired direction. This study was a stepped-wedge cluster randomized trial designed to ascertain the effect of education (recorded online presentation and 2 webinars) and clinical decision support on antibiotic prescribing for acute sinusitis. Both provider education and the clinical decision support intervention improved stewardship and changed diagnostic patterns but the effect of education was brief. Provider education, evaluated as a time effect, led to a significant decrease in antibiotic prescribing (aOR, 0.51; 95% CI, 0.46–0.57) but this effect was not sustained. The authors conclude that education alone is unlikely to have a sustained effect on antibiotic prescribing.

In addition, two uncontrolled studies also found evidence of some positive behaviour change.^13,28^ Armas Freire *et al*.^13^ compared e-learning with face-to-face learning for ICU and internal medicine physicians in Ecuador. They used a quasi-experimental approach with switched learning groups. They measured self-perception of knowledge, attitudes and practices before and after the intervention and also engaged two experts in infectious disease to review medical records to determine antibiotic appropriateness. They found both forms of education improved knowledge, attitudes and practice, with no significant difference between online and face-to-face learning. Expert review of 257 cases revealed statistically significant improvement in prescribing practice for all conditions except bacteraemia, with overall prescribing levels also significantly reduced albeit not consistently across conditions.

Kandeel *et al*.^28^ was an uncontrolled pre-post intervention study focused on an entire district in Egypt. This included education targeted at physicians and pharmacists, as well as some wider public health promotion for the general public. A survey of healthcare professionals was conducted pre- and post-intervention to measure knowledge, attitudes and practices. Mean knowledge and attitude scores supporting the appropriate use of antibiotics improved for both physicians and pharmacists (from 3.8 ± 0.5 to 4.0 ± 0.7 for physicians and from 3.3 ± 0.9 to 4.0 ± 1.2 for pharmacists). There was also a comparison of overall prescribing rates pre- and post-intervention undertaken, with them finding 25% decrease overall in antibiotic prescribing post-intervention for children from 82.1% to 61.5%, and a 22% decrease overall in antibiotic prescribing for adults from 86.7% to 67.9%.

#### Change on the antecedents of behaviour

Hoa *et al*.^26^ conducted a two-armed randomised controlled trial, with medium risk of bias, using a multi-faceted intervention targeting healthcare provider AMS knowledge and practice in Vietnam. Questionnaires completed pre- and post-intervention tested knowledge and practice in case study scenarios, with prescribing practice assessed through an analysis of antibiotic prescribing via completed prescription forms. Total knowledge scores increased statistically in the intervention group (mean improvement 1.17, with the control group also improving albeit less (mean improvement 0.48). Univariate analysis (Wilcoxon) found the mean total knowledge score improved significantly in the intervention group in all subgroups, except in practitioners aged 40–49 years old and those with university education. Prescribing practice outcomes were inconsistent, with the multilevel logistic regression not finding consistent improvement for the intervention condition.

Six other studies found effects on the antecedents of behaviour.^17,18,20,27,40,52^ All were quasi-experimental uncontrolled pre-post intervention designs with knowledge, attitudes and practice (KAP) measured through a questionnaire completed before and after the educational interventions. No study examined longer-term effects, and all used the same KAP measure pre- and post-intervention.

#### No evidence of effect on behaviour or antecedents of behaviour

It is also important to report that two randomised controlled studies, with medium risk of bias, found no evidence of the effectiveness of their respective education interventions on behaviour or the antecedents of behaviour. Both Schwartz *et al*.^47^ and Yasein *et al*.^56^ conducted RCTs with behaviour change as an outcome measure but found no positive effect on prescribing error or prescribing rate, respectively. Indeed, Yasein *et al*.^56^ had improvement on only one aspect of an attitude change measure but found prescribing practice in the intervention group became significantly higher than the control post-intervention. However, both studies had serious design flaws. Schwartz *et al*.^47^ admitted problems with the reliability of their computer assisted prescribing error adjudication process and group allocation problems. Yasein *et al*.^56^ was seriously compromised due to unit closure as a result of the COVID-19 pandemic.

#### Patterning of effectiveness by mode of education

It is not possible to determine patterning by mode of education with any confidence given the heterogeneity of education interventions and lack of clarity about the type of education utilised within some studies. This is particularly problematic in the context of this set of studies having a relatively high risk of bias. It was also not possible to determine a clear effect regarding the qualifications and training of the educators. Not all studies specified who delivered the training, but among those that were most successful most training was delivered, in part or whole, by highly qualified healthcare professionals that matched the target audience profession. However, some tentative reflections on the interventions collated in this review are possible based on those studies that are most effective in changing behaviour and also at low risk of bias.

McNulty *et al*.^36^ is notable for being at low risk of bias and producing clear evidence of behaviour change. This study operated at practice rather than individual level and utilised a complex multi-faceted education intervention involving presentations, reflections on data, promotion of resources, consideration of clinical scenarios and action planning. There were also additional resources provided to accompany the training including leaflets, audit toolkits and a guidance document. The training was not only grounded in evidence based best practice, from Public Health England and the Royal College of General Practitioners, but also drew on psychological theory in order to target attitudes, social norms and perceived barriers to appropriate prescribing behaviour. The educational component was delivered by trained health professionals already working locally (general practitioner, microbiologist or medicines manager). Furthermore, while the intervention clearly involved formal learning,^10^ they also provided a framework for more informal learning, in which the further accumulation of knowledge and change in practice, might occur outside the formal educational intervention.

The other studies that demonstrated clear or some positive effect on behaviour change all utilised substantial training courses, delivered by highly qualified medical professionals^13,19,28^ with the exception of Sharp *et al*.^49^ Sharp *et al*.^49^ was unusual in examining longer-term effects, being a stepped wedge design, but only employed a very simple education intervention consisting of a recorded online presentation and two webinars, with no information about who delivered the sessions. This was still highly effective in the short-term but not the longer term. We do not know how much of this effect was due to an educational intervention alone being insufficient to effect long-term behaviour change in this context or simply the specific design of this intervention.

## Discussion

### Main findings of this study

This systematic review provides a detailed examination of the effectiveness of post-qualification educational interventions designed to enhance antimicrobial stewardship through changed attitudes, increased knowledge and/or changed practice. We have also investigated whether there is evidence demonstrating what works and for whom when considering the type of educational intervention and profession. The findings present a complex picture representing the tendency to incorporate formal education with other interventions, heterogeneity of studies included in this review, and large number of studies at risk of bias conducted on this topic.

Overall, there is evidence of post-qualification (formal) education alone being effective in changing behaviour or the antecedents of behaviour for AMS. This also appears consistent across low- and middle-income countries (LMICs) and high-income countries, with studies in this review split almost 50:50 between high-income and LMICs. That said, the majority of studies reviewed were not controlled, with a very high number of uncontrolled pre-post test designs with increased knowledge or attitudes as the outcome measure. It is also worth noting that while three controlled studies,^26,36,49^ along with the vast majority of uncontrolled studies, found formal education interventions effective in changing behaviour or the antecedents of behaviour for AMS, two RCTs failed to find a positive effect on behaviour or the antecedents of behaviour,^47,56^ albeit both with compromised designs.

There is however a serious lack of evidence regarding the long-term effects of formal education interventions, with few studies conducting longer term follow-up of intervention effects. And where studies were designed to examine longer-term effects, the evidence suggests that formal education alone is unlikely to have a sustained effect on behaviour change. For instance, Sharp *et al*.,^49^ using a stepped wedge design, found formal education to produce a substantive drop in prescribing immediately after the intervention but one which was not sustained beyond a few months, suggesting that changing clinical practice long-term is difficult and may require more than formal education alone. This study did however deploy a very simple educational intervention—an online presentation and two webinars—with no links to psychological or pedagogical theory. When these findings are set against those studies at low risk of bias that were effective in producing clear behaviour change, alongside the large body of uncontrolled pre-post studies, we might tentatively suggest that those education interventions, delivered by trained educators or healthcare professionals, that consist of complex multi-faceted components, notably including recourse to psychological theory, and action planning might be better at producing longer-lasting change. That is, even the simplest of interventions look able to produce short-term change in either behaviour or the antecedents of behaviour but more sophisticated designs, whether alone or in combination with other forms of intervention, may be necessary for effecting longer-term change.

It is also clear from this review that there is insufficient evidence about what works for whom in this specific context of post-qualification education interventions. Education interventions are heterogeneous across studies, often insufficiently specified, and rarely draw upon appropriate psychological or pedagogical theory and evidence in their design. This is in spite of now relatively longstanding concerns being expressed about a general lack of knowledge about optimal scope and intensity of such interventions and the need for further research on this issue for improved intervention design.^47^ There is some evidence that those formal educational interventions that invoke deep learning through complex multifaceted learning experiences, and including opportunities for informal learning, appear most effective, as well as those interventions that seek to embed learning in systemic change, but this must be considered a tentative finding at best. And, of course, interventions that combine formal and informal learning while potentially more effective are also invariably lengthier and so inherently more costly to deliver.

The tendency of high-quality controlled studies to include formal education alongside other (informal learning) interventions—such as audit and feedback—complicates our understanding of whether formal education alone is effective or not in this or other specific contexts. This is particularly problematic when the type of education intervention is also not adequately specified, grounded in extant theory and research or separately evaluated in studies. This approach may however suggest an appreciation, whether explicit or not, of the need for educational interventions to be designed such that they might be embedded within existing practice and facilitate further informal learning.^10^ Furthermore, one controlled study in our review found formal education combined with audit and feedback to be more effective in changing behaviour than formal education alone.^19^ When this is combined with evidence from high-quality studies that demonstrate formal education combined with other interventions to be effective, which were excluded from this study,^58–65^ it is difficult to justify interventions that focus on formal education alone when working within necessary resource constraints unless they are part of a dedicated trial assessing the efficacy of educational interventions.

### Findings in relation to other research

#### Multi-component interventions

Educational interventions are commonly implemented concomitantly with other interventions (e.g. audit and feedback).^66^ Indeed, there is strong evidence from eight reviews that multi-component educational interventions that target both clinicians and patients/the public are effective at reducing antibiotic prescribing for self-limiting conditions.^58–65^ However, multi-component interventions make it difficult to discern which aspects of the intervention contribute the most to the intended outcomes (e.g. changes in antibiotic prescribing). Our review excluded papers where the educational components were not independently evaluated from other parts of the intervention in order to determine the effectiveness of education alone on AMS. This allows us to ascertain whether education alone is effective, as well as appraise what type of education, mode of delivery, medium etc. are helpful in bringing about positive AMS-related change in the workplace.

The Cochrane review by Arnold and Straus^58^ found the effectiveness of an intervention on antibiotic prescribing is dependent on the specific context and barriers to change. They argue strongly that it is not possible to recommend a generic intervention for all behaviours in any setting and that there is a pressing need for further research exploring which components are most effective, hence this study focused on post-qualification education. Our findings were also in line with Arnold and Straus^58^ with regard to the claim that more complex educational interventions, focused on multiple components, were found to be more successful. Indeed, Arnold and Straus^58^ found they were the only interventions with effect sizes of sufficient magnitude to reduce the incidence of antibiotic-resistant bacteria.

#### Type of educational intervention and target learner group

Few studies have sought to understand the type or efficacy of educational interventions for AMS at the professional level. While previous studies have focussed on undergraduates,^67^ fewer have been focussed on those who are qualified, and the extent to which formal (and informal) education is implemented in clinical practice. This is in spite of now aging but then urgent calls for a review of educational interventions in order to fulfil UK and European AMS action plans.^68^ Education interventions are commonly directed towards prescribers (i.e. physicians) and not to other health professionals who play a key role in stewardship e.g. pharmacists, nurses, laboratory professionals.^66,67^ Our review encompassed a broad range of health professionals to determine what strategies may work across different groups in post-qualification education and it was clear that there has been work seeking to improve AMS among a wide variety of health professionals in this context. This is pleasing to see. Behaviour change in the form of changed prescribing practice understandably remains most often focused on physicians, with the antecedents of behaviour change—knowledge, in particular—more commonly the focus with nurses, pharmacists and other non-prescribing healthcare professionals. A wide variety of educational interventions have been shown to improve antimicrobial prescribing practices and infection control in our review yet changing behaviour long-term may well be difficult.

A positive finding with regard to learner group is the way studies are patterned equally across LMICs and high-income countries. Post-qualification education for AMS does not appear to be a field dominated only by researchers and practitioners in high-income countries. That said, learner experience will still be patterned by culture, with intervention design needing to be informed by needs determined within specific countries or regions. The resource limitations in LMICs, for instance, may necessitate different learning design to that in high income countries, albeit we lack the necessary knowledge to know what type of educational intervention might be most effective and in what circumstances.

The low use of behavioural, psychological or other appropriate disciplinary theory to underpin intervention design among the studies reviewed herein is a concern. Motivating and engaging practitioners to effect change will undoubtedly be important in education interventions. Theory is also vital if we are to utilise appropriate forms of education such that it has the potential to effect positive change in attitudes and social norms. The UK National Institute for Health and Care Research and Medical Research Council stress the importance of underpinning theory as a key stage in developing complex interventions.^69^ And, of course, when theory is used to underpin intervention design it needs to be appropriate and utilised correctly.^70,71^ Our findings suggest considerable scope to improve the development (and evaluation) of post-qualification education for AMS through the judicious use of appropriate theory.

### Strengths and limitations

This study provides evidence of best practice with regard to post-professional educational interventions for AMS based on a rigorous search and systematic review process. While the heterogeneity of studies precluded a meta-analysis, we have sought to evaluate the evidence on the basis of a sensitivity analysis and analysis of effectiveness, within the context of a narrative synthesis. Although there have been previous reviews of educational interventions for AMS,^58–65^ this is the first systematic review of effectiveness for post-professional educational interventions for AMS. This is a necessary first step in developing future interventions that are more effective such that we better know what works—that is, which specific types of education intervention are effective—and for whom—that is, which type of formal educational intervention work best for which type of AMS professional. Our analysis provides insight into the present state of intervention science in this specific context, what has worked and what therefore might be repeated and/or investigated and developed further. It also provides a unique and valuable contribution to the literature.

There are limitations, however. First, it was not possible to conduct a meta-analysis given the heterogeneity of studies and our measure of effectiveness must be treated cautiously since this is a relative measure. The effectiveness assessment is built on a body of knowledge that is itself not all at low risk of bias and is therefore not equivalent to a meta-analysis of high-quality controlled studies that may result in more rigorous evidence-based guidance. Our recommendations must therefore be treated with caution. There was considerable heterogeneity in study design, intervention design and outcome measures, all of which problematise the ability to accurately build cumulative knowledge regarding the effectiveness of post-professional education interventions designed to improve AMS. Second, more generally, we are reliant on generally low quality of evidence and the incorporation of some non-controlled studies increases risk of bias in our reporting. While we limited more detailed analysis of effectiveness only to those studies meeting EPOC criteria or an 80% threshold in the MMAT, this does still mean the inclusion of studies with a moderate risk of bias. Third, we must recognise the challenges inherent in defining the inclusion terms for this review. Education is difficult to define. Our decision to adopt a tight definition and exclude studies that did not meet this criterion does mean omission of relevant studies.

### Recommendations for future research

The findings from this investigation suggest a need for a programme of activity to evaluate educational interventions in detail, separate from more generic AMS interventions, such that we can establish which are the effective components within formal education interventions and what works for whom. That is, we need to understand which specific components of education are effective and at what cost, as education may consist of anything from a one-off online lecture to a lengthy face-to-face learning experience. The cost of different educational interventions needs to be weighed against what is most effective. We also need to ascertain what components are effective for which specific population as once again it is likely that different professional groups have different learning preferences and needs. There is also a pressing need for studies that evaluate the long-term effects of such interventions and explore how educational interventions may be designed to best facilitate sustained positive behaviour change or at least how they might most effectively underpin complementary or alternative long-term AMS strategies. Changing established practice is difficult and while behaviour change is clearly feasible through well-designed work-based learning, present evidence suggests the effects of formal educational interventions are short-term, with long-term change a continuing challenge.

## Conclusions

Education continues to be a key component within interventions targeted at improving antimicrobial stewardship. And while education has been studied previously in the context of initial training for professionals,^67^ there has been considerably less focus on post-qualification education. It is important therefore that we seek to understand whether work-based education is effective and more specifically what type of education works best in this context, and for which type of healthcare professional. Our work has provided an in-depth examination of this issue through a systematic review and effectiveness analysis of work focused on post-qualification antimicrobial stewardship. While there is some evidence that education alone is effective in improving AMS, there is limited confidence that this will be sustained. The present evidence base also precludes confidence in knowing what types of educational intervention are most effective and how this might be patterned across type of healthcare professional. We tentatively suggest that educational interventions, informed by psychological or pedagogical theory, that target deep learning and incorporate mechanisms within the intervention for embedding change may well be more effective but further research is needed if we are to make the best and most cost-effective use of education within future AMS intervention design.
